# Validating Driver Behavior and Attitude Measure for Older Italian Drivers and Investigating Their Link to Rare Collision Events

**DOI:** 10.3389/fpsyg.2019.00368

**Published:** 2019-02-21

**Authors:** Giuseppina Spano, Alessandro O. Caffò, Antonella Lopez, Luca Mallia, Michael Gormley, Marco Innamorati, Fabio Lucidi, Andrea Bosco

**Affiliations:** ^1^Department of Education Science, Psychology, Communication Science, University of Bari Aldo Moro, Bari, Italy; ^2^Department of Movement, Human and Health Sciences, Foro Italico University of Rome, Rome, Italy; ^3^School of Psychology, Trinity College Dublin, Dublin, Ireland; ^4^Department of History, Cultural Heritage, Education and Society, University of Rome Tor Vergata, Rome, Italy; ^5^Department of Psychology of Development and Socialization Processes, Sapienza University of Rome, Rome, Italy

**Keywords:** driver behavior questionnaire, self-regulation, attitudes toward traffic, older drivers, confirmatory factor analysis, count data

## Abstract

The present study aimed to: (a) validate the factor structures of three scales assessing driving behavior, attitudes toward traffic safety (ATTS) and self-regulation in driving, in a sample of Italian older adults, through confirmatory factor analyses and (b) to determine the effectiveness of these measures in predicting the likelihood and the frequency of collision involvements in the following year. A 28-item driver behavior questionnaire (DBQ), a 16-item ATTS, a 21-item extended driving mobility questionnaire (DMQ-A) were administered to 369 active Italian drivers, aged between 60 and 91 years. Results showed a four-factor structure for the DBQ, a five-factor structure for the ATTS and a two-factor structure for the Extended DMQ-A, as the best fitting models. Hurdle model analysis of count data with extra-zeros showed that all factors of DBQ predicted the likelihood of road collisions. Risky behavior, except for aggressive violations, self-regulation and attitudes toward traffic rules were associated with the frequency of collision involvement. The aforementioned three scales seemed to be a useful and concise suite of instruments assessing risky as well as protective factors of driving behavior in elderly.

## Introduction

There were 1.25 million road traffic deaths globally in 2013 (World Health Organization [WHO], 2015). Because the global population is gradually aging, older drivers, especially because of their age-related frailty, are likely to make up an increasing proportion of fatality statistics. Sometimes, it is necessary to require the cessation of driving in older people because of sensorial, physical and cognitive age-related deterioration that affects driving ability and leads to an increase in collision probability (Anstey et al., 2005). However, having a driving license and using a car leads to the maintenance of a high level of social and physical functioning among the elderly (Edwards et al., 2009). For instance, in a recent review, Chihuri et al. (2016) showed that the cessation of driving activity in a sample of drivers aged 55 and older, caused various health problems, particularly related to depressive symptoms. Given the importance of these two issues it is important to understand how psychological variables are linked to collision involvement. In a study by Ulleberg and Rundmo (2003), the authors generated a model which proposed personality traits (i.e., aggression, altruism, anxiety, sensation seeking, and normlessness), attitudes toward traffic safety (ATTS) and risk perception as predictors of risky driving behavior. Results showed that personality traits primarily have an effect on risk-taking behavior through the influence of attitude toward traffic safety as a mediator. More relevantly, Lucidi et al. (2014) confirmed the model in a sample of older Italian drivers. In general, novice drivers showed more difficulty in self-regulation, in terms of driving avoidance, than older drivers (Moták et al., 2014). Nonetheless, Gwyther and Holland (2012) suggested that younger and older drivers reported higher score for self-regulation than middle-years’ drivers. According to the authors, these data could be affected by the perception about the driving expertise (i.e., low for younger drivers) and the cognitive functions (i.e., low for older drivers). Besides a wide interest in the theoretical study of risky driving behavior correlates, there is a great concern in developing assessment tests able to identify the relationship between psychological characteristics and probability of being involved in road traffic collision. The driver behavior questionnaire (DBQ – Reason et al., 1990) represents the prominent self-reported assessment tool of risky driving behavior, in terms of violations, errors and lapsus, and has shown to be highly reliable in the accident prediction (de Winter and Dodou, 2010). However, other self-reported behavioral components of the assessment, such as, the attitudes toward traffic rules (e.g., Ulleberg and Rundmo, 2003), and the driving self-regulation (e.g., Owsley et al., 1999), has shown to have an important role in the prediction of road accidents, and they could integrate and support the assessment through the DBQ scale. The three tests presented in this study represent an attempt to provide valid and reliable tools for the assessment of risky driving behavior, ATTS and self-regulation/inhibitory behaviors in the older Italian population, in order to verify which specific behavioral and attitude aspects can contribute to further improve the reliability of a global and general assessment in predicting the likelihood and the frequency of traffic accidents in the elderly population.

### The Driver Behavior Questionnaire (DBQ)

The DBQ is the most used evaluation test on aberrant driving behavior. The original version by Reason et al. (1990), dates back to investigated three dimensions of aberrant driving behavior, namely, *violations*, *dangerous errors*, and *lapses*. A few years later, Parker et al. (1995) confirmed the three-factor structure. It is worth emphasizing that, despite a wide literature which considered the DBQ as the main tool for the evaluation of risky driving, it may be complex to connect the different studies because of the variety of versions used. A wide range of DBQ versions can be found, e.g., a 104-item version by Aberg and Rimmö (1998), a 28-item version (Mattsson, 2012, 2014), and a 9-item version, edited by Martinussen et al. (2013), consisting of the items with the highest factor loadings of the original version of DBQ. The most cited factorial structures seem to be those showing three factors, confirming the original formulation of Reason et al. (1990) and a four-factor solution, proposed by Aberg and Rimmö (1998). It is worth noting that, besides these simple factorial solutions, more complex ones have also been proposed, e.g., Rowe et al. (2015). They proposed a bifactor model of DBQ, including a general factor, which all items load onto, and four latent factors, i.e., *aggressive violations, ordinary violations, slips*, and *errors.* The DBQ has also been used in different cultural context, such as among samples of British, Finnish, and Dutch drivers (Lajunen et al., 2004) and among samples of Irish and Finnish drivers (Mattsson et al., 2015). Smorti and Guarnieri (2016) recently validated the DBQ in an Italian sample aged between 18 and 41 years. They used a 27-item version of the DBQ and found four first-order factors and two second-order factors. Alternatively, Lucidi et al. (2010) confirmed the three-factor model, as in Reason et al. (1990) using a 28-item DBQ, as originally developed by Lawton et al. (1997), on a large Italian sample of young drivers. The same three-factor structure was confirmed in two subsequent studies of older drivers (Lucidi et al., 2014) and professional bus drivers (Mallia et al., 2015). Despite the different ways in which the DBQ has been used, clarification has been provided in terms of its ability to predict involvement in a road traffic collision. In a highly cited meta-analysis, de Winter and Dodou (2010) considered 174 studies using the DBQ, excluding those in non-English language, and showed the predictive power of *errors* and *violations* on self-reported accidents. Subsequently, the authors published an update (de Winter et al., 2015), to provide further information on DBQ’s validity with regard to predicting collisions. The authors confirmed previous findings regarding the preeminent role of errors and violations, especially of speed limits, in predicting self-reported accidents. Furthermore, the authors showed that the DBQ had a strong link also with the recorded violations, demonstrating the reliability of the scale. A recent re-meta-analysis (Af Wåhlberg et al., 2015) identified a number of methodological biases inherent in DBQ research, which led the authors to take a careful approach when interpreting its results. They confirmed the correlation between self-reported errors and violations and collision involvement, but that the correlations should be interpreted in the light of various methodological, statistical and dissemination biases (e.g., systematic measurement error and non-publication of negative results), and the need to take account of other features, such as driving exposure. Certainly, a self-reported evaluation of driving behavior cannot be addressed without the DBQ since it remains the most popular and used tool in traffic psychology. However, it would be interesting to expand self-reported evaluation with other behavioral components, such as attitude and self-regulation, which we will discuss in later sections.

### Attitudes Toward Traffic Safety Scale (ATTS)

The association between attitudes and behavior has been explained by theory of planned behavior (TPB) (Ajzen, 1988, 1991). According to this theory, behavior is co-determined by intentions and by perceived behavioral control; the intentions are the summary of people’s motives, while the perceived behavioral control reflects the perceived ease or difficulty in enacting certain behavior. Subsequently, a meta-analysis (Kraus, 1995) clarified the relationship between behavior and attitude, suggesting that the latter is a strong predictor of the former. In relation to driver behavior, Iversen and Rundmo (2004) analyzed the relationship between attitudes, behavior and involvement in collisions through a survey on a sample of 2614 Norwegian drivers. Their scale has 16 items, on a five-point scale ranging from 1 “strongly agree” to 5 “strongly disagree” to examine the ATTS issues and a 24-items scale to assess risky behaviors. The authors also recorded the number of collisions and near collisions that occurred. Confirmatory factor analysis confirmed a three-factor structure made up of Attitude toward rule violations and speeding, Attitude toward the careless driving of others and Attitude toward drinking and driving. Subsequently, the authors proposed a model involving the factors related to attitudes, those resulting from the analysis of the 24 items of risky behavior and the number of self-reported collisions and found that attitudes contributed to the prediction of self-reported risky behavior. In line with the approach adopted here, the authors encouraged the consideration of other factors beyond attitudes which contribute to collision involvement.

### The Driving Mobility Questionnaire (DMQ-A)

Self-regulation of driving behavior depends on self-monitoring and, subsequently, on the need to change driving behavior should ability change, in order to maintain an acceptable level of safety (Baldock et al., 2006). As in the case of DBQ test, the history of measurement of self-regulation in driving is characterized by the use of a multiplicity of scales, with different numbers of items each corresponding to a potentially dangerous driving activity. Arguably the variability in the measures used has been contributes to by confusion around what constitutes self-regulation of driving behavior. In a recent study, Wong et al. (2015) investigated the factor structure of three variants of an item set that have been used to assess older adults’ driving self-regulation, namely, the Driving Habits Questionnaire (DHQ) (Owsley et al., 1999), the driving mobility questionnaire (DMQ-A) (Baldock et al., 2006), and an extended version of DMQ composed of DMQ-A and twelve new items generated by Sullivan et al. (2011). Wong et al. (2015) intention was to develop a more comprehensive scale. The scale, called *Extended Mobility Driving Questionnaire* (Extended DMQ-A) was composed of 21 items, which required the respondents to indicate the frequency with which they avoided driving in certain conditions, such as, at night in the rain, or in foggy condition, rated on a scale ranging from 1 (never avoid) to 5 (always avoid). An exploratory factor analysis (EFA) revealed a two-factor structure, namely “Internal Driving Environment” and “External Driving Environment,” on the basis of the meaning of the items, related to external factors (e.g., weather conditions) or internal to the car (e.g., driving with or without passengers). However, the authors identified the need to conduct further analysis of the instrument using confirmatory factor analysis.

### Aims of the Study

The general aim of the present study was to combine the contribution of the risky behaviors (DBQ scale) with that of driving attitude (ATTS scale) and driving self-regulation (DMQ-A scale) in predicting the likelihood of collision in the year following the assessment in a sample of active older drivers. Specifically, the preliminary aim was to perform a series of confirmatory factor analysis (CFA) on the aforementioned three scales, involving a sample of active older Italian drivers. Tested models were: (a) a three-factor solution, as in the model confirmed by Lucidi et al. (2010) on a sample of young novice drivers aged between 18 and 23 years, and a four-factor solution, as in Stephens and Fitzharris (2016), for the DBQ scale; (b) a two-factor solution for DMQ-A, as reported by Wong et al. (2015); and (c) a three-factor solution, as reported by Iversen and Rundmo (2004) for the ATTS scale. A *data-driven* five-factor solution was also tested for the ATTS given that an Italian validation for the ATTS scale is lacking. The principal aim of the present study was to examine the role of behavior and attitudes in predicting separately the likelihood and the frequency of self-reported car collisions occurred over the year following the assessment through a Negative Binomial Hurdle (HNB) model (Hu et al., 2011; Hosseinpour et al., 2014). The aforementioned approach is particularly suitable whether the outcome is a count variable characterized by a relatively high number of non-occurrences.

## Materials and Methods

### Participants

Data reported here were collected from 369 community-dwelling older drivers from an initial sample of 405 people (see par. Procedure and Materials for the applied exclusion criteria) recruited in the period between October 2015 and March 2016. They also agreed to be interviewed by phone every month for a total of 12 months to gather information about collisions in which they were involved. Of those who participated, 119 were female; they ranged in age from 60 to 91 years (*M* = 71.1, *SD* = 7.3) and their educational experience ranged from 5 to 23 years (*M* = 9.8, *SD* = 4.4). Each participant had the general aim of the research explained (specific hypotheses were omitted) and was required to provide informed consent to participate. The study was approved by the local ethical committee and was performed in accordance with the Helsinki Declaration and its later amendments or comparable ethical standards.

### Procedure and Materials

Participants were interviewed in order to provide a range of demographic information including age, gender, education, as well as clinical history and current health status. Moreover, for the whole sample, the number of occasions of driving (less than once per month, once or twice per month, at least once a week and more than once a week) in the previous years was recorded. The inclusion criteria for the study were: (a) having a valid car driving license; (b) drive a car at least once per month; (c) absence of visual (uncorrected) and/or physical impairment; (d) no history of cranial trauma, brain lesions, or stroke. The aforementioned data were evaluated through an anamnestic interview. Also, cognitive efficiency has been assessed through the Montreal Cognitive Assessment (MoCA, Nasreddine et al., 2005) where a score higher than 17 is considered as the best threshold to discriminate probable mild cognitive impairment in Italian population (Bosco et al., 2017). Autonomy in the management of daily activities has been assessed through the Activities of Daily Living (ADL, Katz, 1983) and the Instrumental Activities of Daily Living (IADL, Lawton and Brody, 1969). Finally, absence of geriatric depression was evaluated through the Geriatric Depression Scale (GDS_15, Brink et al., 1982). On the basis of these criteria, 36 drivers were excluded from the final sample (exclusion rate 9%). The following versions of the three scales mentioned above were used:

(A) The Italian 28-item version of *Driver Behavior Questionnaire* (DBQ), developed by Lawton et al. (1997), and adapted to the Italian context by Lucidi et al. (2010), rated on a six-point scale ranging from 0 (Never) to 5 (almost always). In this scale, high score indicated a high frequency of aberrant behaviors during driving activities.
(B) The 16-item scale of the *Attitudes Toward Traffic Safety Scale* (ATTSS), developed by Iversen and Rundmo (2004) and translated in Italian by Lucidi et al. (2010), on a five-point response scale ranging from “strongly disagree” (1) to “strongly agree” (5). A high score represented a negative attitude toward traffic safety rules.
(C) The 21-item version of *Driving Mobility Questionnaire* (Extended DMQ-A) by Wong et al. (2015), rated on a scale ranging from 1 (never avoid) to 5 (always avoid). The Italian translation of DMQ-A was created by the authors of the present study. The questionnaire was initially translated into Italian. This version was then given to a translator, fluent in English, who did not know of the existence of the original questionnaire, who was asked to translate the questionnaire back into English. This new English version was then compared to the original English version which proved to be grammatically and semantically equivalent, thus allowing the Italian version to be accepted as the final version of DMQ-A to be used in this study.

Each participant was interviewed by a well-trained research assistant who administered the questionnaire items to the interviewee and marked the answers on the response protocol. The entire procedure including the administration of the preliminary interview/tests to evaluate the inclusion criteria and the three driving questionnaires lasted approximately one and a half hours. A break was granted whenever requested.

### Statistical Analysis

Confirmatory factor analysis (CFA) models were estimated using the R software (R Development Core Team, 2013) and the lavaan package (Rosseel, 2012), and graphically reported using the qgraph package (Epskamp et al., 2012). Internal consistency was determined using Cronbach’s alpha. Confirmatory factor analysis (CFA) were carried out in order to test the most consistent factorial solutions existing in literature and to present the best factorial solution for each scale, namely, a four-factor DBQ solution, a five-factor solution for the ATTS and a two-factor solution for the Extended DMQ-A. The following fit indices and the respective cut-off for goodness of fit have been reported: the Chi-squared value (*χ*^2^), to assess the overall goodness of fit of the model, even if very sensitive to sample size and no longer considered as a basis for acceptance or rejection of the model (Schermelleh-Engel et al., 2003), the Comparative Fit Index (CFI) (a value of CFI ≥ 0.95 is currently considered as indicative of good fit) (Hu and Bentler, 1999), the Tucker Lewis index (TLI) (a cut-off of 0.95 or greater stands for a good model fit), the Root Mean Square Error of Approximation (RMSEA) (a value lower than 0.05 is considered acceptable), and the Standardized Root Mean Square Residual (SRMR) (a value less than 0.08 is considered satisfactory) (MacCallum et al., 1996).

For the CFAs, a parametric method of data analysis has been adopted. In this respect, a variety of parametric, non-parametric and semi-parametric approaches have been explored in literature. Briefly, parametric statistics assumes that data produced by the sample comes from a population that follows a probability distribution based on a fixed set of parameters. An example of parametric method is the *Maximum Likelihood Estimation* who establishes values for the parameters of a model maximizing the probability that the model reflects the observed data (Jöreskog, 1978; Bollen, 2005). Non-parametric statistics do not need data fit with a normal distribution and therefore the model structure is determined from data instead of being specified *a priori*. An example is the *Partial Least Squares* analysis which estimates the latent variables as weighted aggregates (e.g., Lohmöller, 1989). Lastly, it is also worth mentioning the semi-parametric statistics which has both parametric and non-parametric components. Example of semi-parametric models are the *Cox Proportional Hazards* model (Balakrishnan et al., 2004) and the *Generalized Maximum Entropy* for estimating structural equation models (Ciavolino and Al-Nasser, 2009; Ciavolino and Dahlgaard, 2009; Carpita and Ciavolino, 2017).

In addition, predictive validity of each factor was assessed, by determining which factors predict collision involvement in the following year. A hurdle negative binomial (HNB) model was performed using the “pscl” package (Zeileis et al., 2008), since classical regression models were not appropriate due to the shape of the distribution of the outcome data. Thus, although the use of Poisson models is strongly recommended in the case of count data, it is not with overdispersion – events that are much less likely to occur than the opposite (Gardner et al., 1995). The number of road collisions occurring in a one-year period fits into that category. As far as we know, there are many statistical models that could be considered to represent these data including: negative binomial (NB), zero-inflated Poisson (ZIP), zero-inflated negative binomial (ZINB), Poisson hurdle (HP), and HNB models but Hurdle Models are the most suitable to operate on this type of data (Hu et al., 2011; Hosseinpour et al., 2014). Unlike the zero-inflated model, hurdle models consider the distribution of zero and non-zero separately. They also attribute to zero the actual value of “structural zero,” differently from zero-inflated, which consider the fact that zeroes can also arise from non-exposure to the phenomenon (“sampling zeros”). Given the sample was exclusively composed of active drivers, we can state that each participant is exposed to the risk of a collision. For this reason, Poisson Hurdle Model and HNB model seem to be the most appropriate. Although the two models may look similar, the use of the NBH model is recommended when the observed outcome has an average lower than its variance, as is the case for a crash involvement distribution.

## Results

### Confirmatory Factor Analysis and Reliability of the Three Scales

Table 1 shows the fit indices for the models tested, namely, a three- and a four-factor solution for the DBQ scale, a three- and a five-factor solution for the ATTS, and two two-factor solutions for DMQ-A.

**Table 1:** Fit indices of the model tested.

		Fit indices						
	Model	*χ*^2^	*df*	CFI	TLI	RMSEA	SRMR	AIC
Reason et al., 1990	3 factors (28 items)	664.320	347	0.822	0.806	0.05	0.057	24523.528
Aberg and Rimmö, 1998	4 factors (28 items)	470.256	343	0.929	0.921	0.032	0.048	24337.464
Iversen and Rundmo, 2004	3 factors (16 items)	225.862	101	0.953	944	0.058	0.047	15601.311
Present study	5 factors (16 items)	90.897	94	1.000	1.000	0.000	0.030	15480.346
Wong et al., 2015	2 factors (21 items)	984.686	188	0.772	0.745	0.107	0.111	23689.237
Present study	2 factors (14 items)	192.957	73	0.951	0.939	0.067	0.075	15182.647

**Figure 1:**
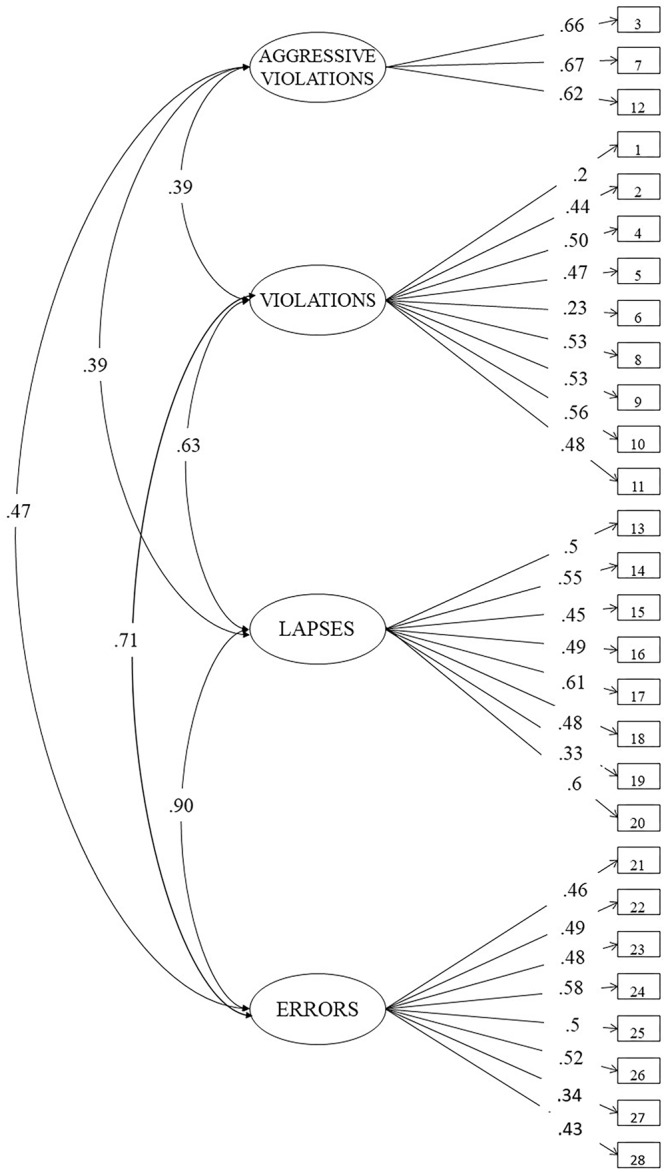
Final model for the 28-item driver behavior questionnaire.

As reported by Stephens and Fitzharris (2016), a four-factor solution (see Figure 1), i.e., *Aggressive Violations* (AV – three items), *Violations* (V – nine items), *Lapses* (L – eight items), and *Errors* (E – eight items) has shown to be the best model for the DBQ. The model exhibited the following indices of goodness of fit: *χ*^2^(343) = 470.256, *p* < 0.001, CFI = 0.929, TLI = 0.921; RMSEA = 0.032; SRMR = 0.048. Internal consistency of each factor and the DBQ total score was also evaluated using Cronbach’s alpha. As a scale, DBQ showed a consistency value of 0.86. In terms of single factors, Aggressive Violations, Violations, Lapses and Errors showed the following values: α = 0.69, α = 0.68, α = 0.73, and α = 0.70, respectively. All the reliability coefficients were close to or exceeded the threshold of α = 0.70.

**Figure 2:**
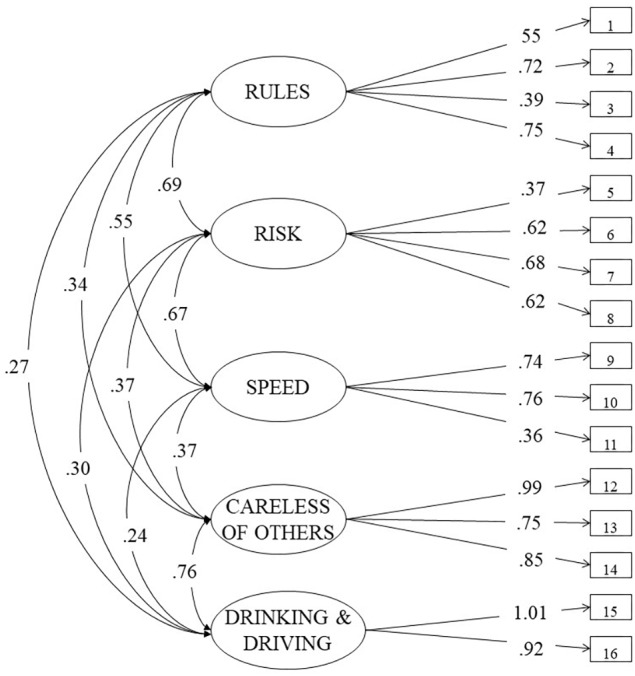
Final model for the l6-item attitudes toward traffic safety.

For the ATTS scale, the best factorial solution was a five-factor solution (see Figure 2) namely, *Rules* (RU – four items), *Risk* (RI – four items), *Speed* (SP – three items), *Careless of others* (CO – three items), and *Drinking and Driving* (D- two items). The model showed the following fit indices: *χ*^2^(94) = 90.897, *p* > 0.5, CFI = 1.000, TLI = 1.000; RMSEA = 0.000; SRMR = 0.030. Cronbach’s alpha for the whole scale was α = 0.85, revealing a satisfactory internal consistency. Rules, Risk and Speed sub-scales showed an acceptable internal consistency, i.e., α = 0.69, α = 0.65, α = 0.63, respectively, whereas, Careless of Others and Drinking and Driving revealed excellent values of α = 0.89 and α = 0.96, respectively.

**Figure 3:**
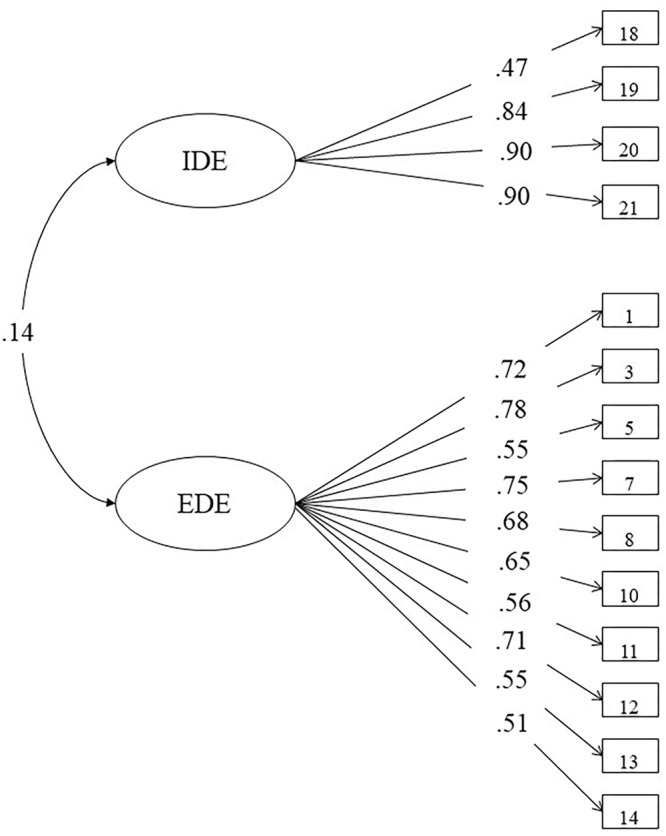
Final model for the 14-item driving mobility questionnaire.

With respect to the DMQ-A, the model estimated revealed a two-factor structure (see Figure 3) with the latent factors labeled External Driving Environment (EDE) and Internal Driving Environment (IDE). Since some factor loadings were inadequate (<0.4), the corresponding items were removed from the model. Consequently, the final version of the scale was composed of 14 items. The seven deleted items were: item 2: “In the rain,” item 4: “Peak hour,” item 6: “High traffic roads,” item 9: “At the start/end of school times,” item 15: “Parallel parking,” item 16: “Right turns,” and finally, item 17: “Roundabouts.” The final 14-item DMQ-A model’s fit indices were as follows: *χ*^2^(73) = 192.957, *p* < 0.001, CFI = 0.951, TLI = 0.939, RMSEA = 0.067, SRMR = 0.075. As for the aforementioned scales, the two latent factors and the total scale showed acceptable internal consistency reliability; in particular EDE, IDE and the total scale’s Cronbach’s alpha values were α = 0.88, α = 0.86, and α = 0.68, respectively.

Table 2 shows the correlations among all the factors’ scales; mean and standard deviation for each factor.

**Table 2:** Correlation matrix of all the variables, mean, and SD.

Factor	AggViol	Viol	Lapses	Errors	EDE	IDE	Rules	Risk	Speed	CO	Mean	*SD*
AggViol											3.024	2.935
Viol	0.258**										5.152	4.705
Lapses	0.271**	0.446**									5.412	4.141
Errors	0.321**	0.469**	0.639**								2.921	3.023
EDE	-0.014	-0.208**	0.074	-0.005							25.924	10.574
IDE	0.069	0.047	0.015	0.060	0.207**						5.349	3.054
Rules	-0.127*	-0.410**	-0.223**	-0.227**	0.157**	0.010					17.076	3.413
Risk	-0.209**	-0.388**	-0.229**	-0.272**	0.121*	0.006	0.469**				15.328	3.799
Speed	-0.139**	-0.312**	-0.204**	-0.192**	0.092	0.102*	0.375**	0.452**			11.501	3.054
CO	-0.067	-0.170**	-0.065	-0.143**	0.070	0.045	0.290**	0.283**	0.299**		10.035	2.432
DD	-0.036	-0.151**	-0.017	-0.061	0.062	0.065	0.232**	0.237**	0.201**	0.724**	3.450	1.527

^∗∗^*Correlation is significant at the 0.01 level (2-tailed). ^∗^Correlation is significant at the 0.05 level (2-tailed)*.

### The Link Between Driver Behavior, Attitude, and Rare Collision Events

Preliminary Chi squared analyses have been conducted to verify the relationship between age/education and collisions and to investigate the role of age and education variables as possible mediators. Given the large sample size and the well-known sensitivity of Chi-square distribution to sample size, we have chosen a conservative *p* < 0.01 as the reference level for statistical significance. Chi square analysis was performed by splitting the sample into two sub-samples according to age (60–74 and 75–91 years) and the median of education (i.e., 8 that corresponds to the achievement of high school graduation in Italy). No statistically significant differences emerged between age [*X*^2^ (2, *N* = 369) = 6.41, *p* = 0.04] and education [*X*^2^(2, *N* = 369) = 3.60, *p* = 0.17] with respect to the outcome, i.e., collision, thus age and education variables have not been considered in the subsequent analysis.

As described previously in the *Statistical Analysis* section, NBH model have the advantage of estimating both the likelihood of engaging in a specific event, that is, the hurdle portion, and the frequency with which that event occurs, that is, the count portion (Arens et al., 2014).

In the present sample, 33 drivers reported one crash over the year (about 8%) while 7 drivers reported 2 (about 2%). Table 3 shows that all the DBQ variables (Violations, Aggressive Violations, Lapses and Errors) are equally associated with the likelihood of engaging in a car collision. In other words, a higher frequency of self-reported aberrant driving behavior predicted the likelihood of having a collision. However, this is not the case for other variables, namely EDE, IDE, Rules, Speed, Risk, Careless of Others, and Drinking and Driving. In fact, it seemed that these variables do not significantly predict the likelihood of having a collision.

**Table 3:** Estimation of the Negative Binomial Hurdle (NBH) model with all factors as independent variables.

	Hurdle model		Count model	
	Estimate	*p*	Estimate	*P*
(Intercept)	-84.975	0.026*	4.693	0.490
Violations	2.753	0.026*	0.855	0.032*
Aggressive violations	2.908	0.026*	-0.737	0.249
Lapses	2.684	0.027*	-0.637	0.003**
Errors	2.054	0.024*	2.617	<0.001***
EDE	-0.028	0.837	-0.649	0.004**
IDE	0.015	0.947	0.901	<0.001***
Rules	-0.422	0.363	-1.134	<0.001***
Risk	-0.437	0.202	-0.462	0.091
Speed	0.294	0.439	-0.044	0.852
Careless of others	0.069	0.842	-0.665	0.428
Drinking and driving	1.101	0.198	1.110	0.216

*Number of collisions is the dependent variable. Signif. codes: ^∗∗∗^0.001, ^∗∗^0.01, ^∗^0.05*.

With respect to frequency (i.e., count model), Aggressive Violations became not significantly associated with the frequency of collisions. While, the other three variables maintained a significant relationship with the outcome. In other words, as the number of Violations and, with a larger extent, the number of Errors increased, the frequency of collision increased as well. An unexpected result relates to the variable Lapses. According to the NBH model, collisions were inversely associated with number of Lapses. Furthermore, both the DMQ-A variables showed to be associated with the frequency of accidents in a year. In particular, a higher self-regulation concerning environmental aspects (EDE) was positively associated with a lower frequency of collisions, while, a higher self-regulation involving the personal, “internal” aspects of risk driving (IDE) was surprisingly associated with a higher frequency of collisions. Moreover, a positive attitude toward traffic rules (i.e., the variable labeled as Rules) was significantly associated with a lower frequency of collision in a year. In conclusion, Errors (DBQ) and Rules (ATTS) showed to be the most relevant predictors of frequency of collisions. Finally, Speed, Risk, Careless of Others and Drinking and Driving were not associated both with likelihood and frequency of car collision.

## Discussion

The first aim of the present study was to assess the factorial validity of three widely used scales on risky driving behavior, positive attitudes toward traffic rules and self-regulation in dangerous driving situations on a sample of Italian older active drivers, namely a 28-item DBQ, a 16-item ATTS and a 21-item Extended DMQ-A. Using confirmatory factor analysis, complied with the four-factor structure found in previous research, the final DBQ model was composed of four latent factors. The four-way distinction of the DBQ has been confirmed with respect to previous findings (e.g., Aberg and Rimmö, 1998; Rimmö, 2002; Bener et al., 2008; Martinussen et al., 2013; Mattsson et al., 2015; Cordazzo et al., 2016). Despite the presence of previous empirical evidence that supported the three-factor structure for the DBQ (e.g., Parker et al., 1995; Lucidi et al., 2014; Mallia et al., 2015), the four-factor solution appears to be the most appropriate in the present sample according to the fit indices. It is worth emphasizing that this is a further subdivision of “driving violations” dimension, which, therefore, does not seem to substantially change the original three-way distinction in violations, lapses and errors among risky driving behaviors.

An interesting result is the high covariance between errors and lapses variables in the CFA model of the DBQ scale. This seems to be in line with the idea that errors and violations are underlined to different cognitive processes. Reason et al. (1990) suggested that errors as well as lapses are unintentional, and the latter are included in the former ones. On the contrary, violations are deliberate infringements of traffic rules, hence intentional. This was later confirmed by Özkan et al. (2006) who argued as a two-factor solution, i.e., errors (composed of lapses, slips, and mistakes) and violations, was the most stable model, over time. On the other hand, other scholars (Lajunen et al., 2004; Smorti and Guarnieri, 2016) suggested a second-order factor model based on errors (including mistakes and lapses) and violations (including general and aggressive violations).

As regards the ATTS scale, the three-factor structure showed very good fit indices and seemed to be consistent with that originally proposed by Iversen and Rundmo (2004) involving a sample of Norwegian middle-aged drivers. Nevertheless, the final choice fell on a five-factor structure, since it provided a better fit to the current data. The final model of DMQ-A scale was composed of two latent factors labeled EDE and IDE, as already suggested by Wong et al. (2015). The lack of an Italian validation requested to follow a *data-driven approach*. In our Italian DMQ-A version, the items 2, 4, 6, 9, 15, 16, and 17 have been removed because of irrelevant factor loading values.

The second aim was to find out which factors of each scale predicted collision involvement over the period of a year. As addressed by several scholars (e.g., Af Wåhlberg et al., 2015; de Winter et al., 2015) the data in literature revealing an association between aberrant behaviors at the wheel (i.e., violations, lapses and errors) and self-reported accidents data may be inflated by several methodological biases, including common method variance effect. In order to overcome this possible bias, the present study introduced a design in which the older drivers were contacted by telephone monthly for a year to register any collision may be occurred. This methodology has two main strong points: (a) introduces a prospective design allowing to explore the predictive capacity of each measure to predict collisions excluding a possible common method variance effect; (b) reduces the possibility of a recall effect, asking older drivers to analyze only a limited time frame (last month).

The results showed that driving violations, lapses and errors strongly affect the risk of collision, while the role of aggressive violations appears to be weaker than the others, as it seems to predict the likelihood of incurring in a collision but not its frequency. These results are in line with the literature in that risky driving in older drivers is positively related to self-reported crash involvement (e.g., Lucidi et al., 2014; Af Wåhlberg et al., 2015).

The results also revealed the significant impact of self-regulation on the frequency of collision between subjects who have already had an accident. Data on the present sample of older drivers showed that high self-regulation with respect to potentially hazardous external situations, such as, adverse weather conditions, are associated with a lower frequency of accidents in drivers who have already had an accident. On the contrary, self-regulating in a potentially risky internal environment, that is, for instance, the presence of children passenger in the car, was associated to a higher frequency of collisions. Indeed, these findings suggest that self-regulating behavior during these situations can even be a risk factor for the drivers and passengers. Self-regulation may be a mediator between other constructs, such as certain personality traits and/or cognitive variables (Devlin and McGillivray, 2016). Indeed, several studies argue that self-regulation is a multidimensional factor, affected by several components, such as decision making (Molnar et al., 2014), self-confidence (Molnar and Eby, 2008), and personality traits, such as attachment style (Gillath et al., 2017). It could also be hypothesized that other personality traits, such as anxiety, may affect self-regulation, especially if we take into account those situations in which the driver feels the responsibility for the safety of other passengers, even more if children. Thus, a cautious explanation of our result might be that a self-reported propensity to self-regulate associated to the presence in the car of other passengers could reveal an anxious personality inclined to implement potentially risky behaviors at the wheel. With respect to the ATTS factors, the analysis shows that only a positive attitude toward traffic rules was associated to the frequency of collision. Conversely, other factors regarding risk avoidance, high speed, caring for the others, and alcohol-driving did not significantly impact both on likelihood and frequency of collisions. Again, a possible explanation may be that ATTS could be dependent on specific personality dimensions, as is the case of the *personality-attitudes-risk driving behavior* model (Ulleberg and Rundmo, 2003). In addition, several studies showed that older drivers are less prone to participate in dangerous behaviors, such as reckless driving (Doroudgar et al., 2017), abuse of alcohol before and during driving (Bates et al., 2014), likely due to concerns over their own fragility, than young car drivers. In summary, it seems that the behavior, and therefore, the actual action, shows its close link with the consequence, that is, the accident. However, once the accident has occurred, other variables may be involved in affecting the likelihood of a relapse. The present results converged on the validity of the DBQ as the preferred tool for the prediction of self-reported accidents, and confirmed, also in the present sample of active older drivers, the strict relationship between attitudes toward safety (i.e., attitudes toward rules, risk and speed) and all the four dimensions of the DBQ. As in previous research (e.g., Lucidi et al., 2014; Mallia et al., 2015) attitudes are more related to ordinary violations than to other driving behaviors. This data is in line with the nature of the ordinary violations that are the results of a deliberate and conscious choice resulting more influenced by attitudes than other aberrant behaviors that are may be more linked to cognitive functioning (i.e., errors and lapses).

The components of the DBQ and self-regulation do not seem to have a direct link, as confirmed by previous findings (Rimmö and Hakamies-Blomqvist, 2002; Gabaude et al., 2010). On the contrary, in the older drivers, the role of attitudes toward respect for the law and the traffic rules seems to be very strong, unlike what happens for young people (Yagil, 1998). It is worthwhile to note that the involvement of other variables, such as self-regulation and attitude toward road safety, can be useful in assessing the likelihood of relapses (Iversen, 2004) and in their prevention, as well as in the prediction of types of accident with respect to different factors of attitudes and self-regulation examined (Slavinskiene et al., 2014). Overall, the relationship between attitudes, self-regulation and behaviors might be more complex than expected and, also be mediated by other factors not considered in the present study. Future studies will have to investigate the complex relationship between cognitive, personality variables and the three constructs under consideration here and how this relationship affects the number of short and long-term risks of being involved in collision.

The present study has some limitations. All the data are self-reported. Despite the monthly interviews with which the research assistants maintained regular contacts with participants, the role of memory deficits or social desirability on accident reporting cannot be ruled out. Despite the fact that Helman and Reed (2015) have argued for a clear association between self-reported and objective measures, when using a driving simulator, accesses to objective data relating to collision involvement would clearly have greater validity. A limitation is also the lack of other objective criteria, beyond the number of accidents, such as, traffic fines. This point is closely linked to the previous limitation, as the authors hypothesized that the participants were not inclined to declare the traffic fines. Future research may use more reliable methods to collect objective criteria, possibly in cooperation with local authorities. Despite the presence of the aforementioned limitations, the present study proposed a contribution to the creation of a suitable driving ability assessment procedure, as suggested by some scholars (e.g., Af Wåhlberg et al., 2015), in a specific and critical sample, namely active older drivers, in order to identify the specific risk and protection factors that act on the likelihood of being involved in risky behavior and collisions. A systematic approach to the assessment and prevention of incorrect driving behaviors could be a step to turn potential victims of traffic injuries into safer drivers. For this reason, it would be desirable to implement personalized educational programs, firstly, for the assistance of drivers at risk of loss of the driving license, and secondly, to amend such risky behaviors ensuring autonomy and functionality as essentials of cognitive reserve (Caffò et al., 2016) of older drivers in a safety way.

## Data Availability

The raw data supporting the conclusions of this manuscript will be made available by the authors, without undue reservation, to any qualified researcher.

## Ethics Statement

This study was carried out in accordance with the Declaration of Helsinki and its later amendments or comparable ethical standards. All subjects gave written informed consent. The protocol was approved by the Local Ethics Committee of the Department of Educational Sciences, Psychology, Communication, University of Bari (nr. 3660-CEL02/17).

## Author Contributions

GS, AC, AL, LM, FL, and AB conceived the original idea and were primarily responsible for the data collection, data analysis, and interpretation of results. GS, AC, and LM were primarily responsible for drafting the manuscript. MG, MI, FL, and AB critically revised the draft of the manuscript and supervised the general process.

## Conflict of Interest Statement

The authors declare that the research was conducted in the absence of any commercial or financial relationships that could be construed as a potential conflict of interest.

## References

[B1] Aberg L. Rimmö P. A. (1998). Dimensions of aberrant driver behaviour. *Ergonomics* 41 39–56. 10.1080/001401398187314 9468806

[B2] Af Wåhlberg A. E. Barraclough P. Freeman J. (2015). The driver behaviour questionnaire as accident predictor; a methodological re-meta-analysis. *J. Safety Res.* 55 185–212. 10.1016/j.jsr.2015.08.003 26683562

[B3] Ajzen I. (1988). *Attitudes, Personality and Behavior.* Milton Keynes: Open University Press.

[B4] Ajzen I. (1991). The theory of planned behavior. *Organ. Behav. Hum. Decis. Processes* 50 179–211. 10.1016/0749-5978(91)90020-T

[B5] Anstey K. J. Wood J. Lord S. Walker J. G. (2005). Cognitive, sensory and physical factors enabling driving safety in older adults. *Clin. Psychol. Rev.* 25 45–65. 10.1016/j.cpr.2004.07.008 15596080

[B6] Arens A. M. Gaher R. M. Simons J. S. Dvorak R. D. (2014). Child maltreatment and deliberate self-harm: a negative binomial hurdle model for explanatory constructs. *Child Maltreat.* 19 168–177. 10.1177/1077559514548315 25189325

[B7] Balakrishnan N. Rao C. R. Rao C. R. (2004). *Handbook of statistics: advances in survival analysis.* Amsterdam: Elsevier.

[B8] Baldock M. R. Mathias J. L. McLean J. Berndt A. (2006). Self-regulation of driving and older drivers’ functional abilities. *Clin. Gerontol.* 30 53–70. 10.1300/J018v30n01_05

[B9] Bates L. J. Davey J. Watson B. King M. J. Armstrong K. (2014). Factors contributing to crashes among young drivers. *Sultan Qaboos Univ. Med. J.* 14:e297–e305. PMC4117653 25097763

[B10] Bener A. Özkan T. Lajunen T. (2008). The driver behaviour questionnaire in arab gulf countries: qatar and united arab emirates. *Accid. Anal. Prev.* 40 1411–1417. 10.1016/j.aap.2008.03.003 18606274

[B11] Bollen K. A. (2005). “Structural equation models,” in *Encyclopedia of Biostatistics* (Atlanta, GA: American Cancer Society). 10.1002/0470011815.b2a13089

[B12] Bosco A. Spano G. Caffò A. O. Lopez A. Grattagliano I. Saracino G. (2017). Italians do it worse. *Aging Clin. Exp. Res.* 29 1113–1120. 10.1007/s40520-017-0727-6 28155182

[B13] Brink T. L. Yesavage J. A. Lum O. Heersema P. H. Adey M. Rose T. L. (1982). Screening tests for geriatric depression. *Clin. Gerontol.* 1 37–43. 10.1300/J018v01n01_06 7183759

[B14] Caffò A. O. Lopez A. Spano G. Saracino G. Stasolla F. Ciriello G. (2016). The role of pre-morbid intelligence and cognitive reserve in predicting cognitive efficiency in a sample of Italian elderly. *Aging Clin. Exp. Res.* 28 1203–1210. 10.1007/s40520-016-0580-z 27149863

[B15] Carpita M. Ciavolino E. (2017). A generalized maximum entropy estimator to simple linear measurement error model with a composite indicator. *Adv. Data Anal. Classif.* 11 139–158. 10.1007/s11634-016-0237-y

[B16] Chihuri S. Mielenz T. J. DiMaggio C. J. Betz M. E. DiGuiseppi C. Jones V. C. (2016). Driving cessation and health outcomes in older adults. *J. Am. Geriatr. Soc.* 64 332–341. 10.1111/jgs.13931 26780879 PMC5021147

[B17] Ciavolino E. Al-Nasser A. D. (2009). Comparing generalised maximum entropy and partial least squares methods for structural equation models. *J. Nonparametr. Stat.* 21 1017–1036. 10.1080/10485250903009037

[B18] Ciavolino E. Dahlgaard J. J. (2009). Simultaneous equation model based on the generalized maximum entropy for studying the effect of management factors on enterprise performance. *J. App. Stat.* 36 801–815. 10.1080/02664760802510026

[B19] Cordazzo S. T. Scialfa C. T. Ross R. J. (2016). Modernization of the driver behaviour questionnaire. *Accid. Anal. Prev.* 87 83–91. 10.1016/j.aap.2015.11.016 26655522

[B20] de Winter J. C. Dodou D. Stanton N. A. (2015). A quarter of a century of the DBQ: some supplementary notes on its validity with regard to accidents. *Ergonomics* 58 1745–1769. 10.1080/00140139.2015.1030460 25777252

[B21] de Winter J. C. F. Dodou D. (2010). The driver behaviour questionnaire as a predictor of accidents: a meta-analysis. *J. Safety Res.* 41 463–470. 10.1016/j.jsr.2010.10.007 21134510

[B22] Devlin A. McGillivray J. (2016). Self-regulatory driving behaviours amongst older drivers according to cognitive status. *Transp. Res. Part F* 39 1–9. 10.1016/j.trf.2016.02.001

[B23] Doroudgar S. Chuang H. M. Perry P. J. Thomas K. Bohnert K. Canedo J. (2017). Driving performance comparing older versus younger drivers. *Traffic Inj. Prev.* 18 41–46. 10.1080/15389588.2016.1194980 27326512

[B24] Edwards J. D. Lunsman M. Perkins M. Rebok G. W. Roth D. L. (2009). Driving cessation and health trajectories in older adults. *J. Gerontol. Series A* 64 1290–1295. 10.1093/gerona/glp114 19675177 PMC2773808

[B25] Epskamp S. Cramer A. O. Waldorp L. J. Schmittmann V. D. Borsboom D. (2012). Qgraph: network visualizations of relationships in psychometric data. *J. Stat. Softw.* 48 1–18. 10.18637/jss.v048.i04

[B26] Gabaude C. Marquié J. C. Obriot-Claudel F. (2010). Self-regulatory driving behaviour in the elderly: relationships with aberrant driving behaviours and perceived abilities. *Le Trav. Hum.* 73 31–52. 10.3917/th.731.0031

[B27] Gardner W. Mulvey E. P. Shaw E. C. (1995). Regression analyses of counts and rates: poisson, overdispersed poisson, and negative binomial models. *Psychol. Bull.* 118 392 10.1037/0033-2909.118.3.392 7501743

[B28] Gillath O. Canterberry M. Atchley P. (2017). Attachment as a predictor of driving performance. *Transp. Res. Part F* 45 208–217. 10.1016/j.trf.2016.12.010

[B29] Gwyther H. Holland C. (2012). The effect of age, gender and attitudes on self-regulation in driving. *Accid. Anal. Prev.* 45 19–28. 10.1016/j.aap.2011.11.022 22269481

[B30] Helman S. Reed N. (2015). Validation of the driver behaviour questionnaire using behavioural data from an instrumented vehicle and high-fidelity driving simulator. *Accid. Anal. Prev.* 75 245–251. 10.1016/j.aap.2014.12.008 25528197

[B31] Hosseinpour M. Yahaya A. S. Sadullah A. F. (2014). Exploring the effects of roadway characteristics on the frequency and severity of head-on crashes: case studies from Malaysian Federal Roads. *Accid. Anal. Prev.* 62 209–222. 10.1016/j.aap.2013.10.001 24172088

[B32] Hu L. T. Bentler P. M. (1999). Cutoff criteria for fit indexes in covariance structure analysis: conventional criteria versus new alternatives. *Struct. Equ. Modeling* 6 1–55. 10.1080/10705519909540118

[B33] Hu M. C. Pavlicova M. Nunes E. V. (2011). Zero-inflated and hurdle models of count data with extra zeros: examples from an HIV-risk reduction intervention trial. *Am. J. Drug Alcohol Abuse* 37 367–375. 10.3109/00952990.2011.597280 21854279 PMC3238139

[B34] Iversen H. (2004). Risk-taking attitudes and risky driving behaviour. *Transp. Res. Part F* 7 135–150. 10.1016/j.trf.2003.11.003

[B35] Iversen H. Rundmo T. (2004). Attitudes towards traffic safety, driving behaviour and accident involvement among the Norwegian public. *Ergonomics* 47 555–572. 10.1080/00140130410001658709 15204303

[B36] Jöreskog K. G. (1978). Structural analysis of covariance and correlation matrices. *Psychometrika* 43 443–477. 10.1007/BF02293808

[B37] Katz S. (1983). Assessing self-maintenance: activities of daily living, mobility, and instrumental activities of daily living. *J. Am. Geriatr. Soc.* 31 721–727. 10.1111/j.1532-5415.1983.tb03391.x 6418786

[B38] Kraus S. J. (1995). Attitudes and the prediction of behavior: a meta-analysis of the empirical literature. *Pers. Soc. Psychol. Bull.* 21 58–75. 10.1177/0146167295211007 17515779

[B39] Lajunen T. Parker D. Summala H. (2004). The manchester driver behaviour questionnaire: a cross-cultural study. *Accid. Anal. Prev.* 36 231–238. 10.1016/S0001-4575(02)00152-5 14642877

[B40] Lawton M. P. Brody E. M. (1969). Assessment of older people: self-maintaining and instrumental activities of daily living. *Gerontologist* 9 179–186. 10.1093/geront/9.3_Part_1.179 5349366

[B41] Lawton R. Parker D. Manstead A. S. Stradling S. G. (1997). The role of affect in predicting social behaviors: the case of road traffic violations. *J. App. Soc. Psychol* 27 1258–1276. 10.1111/j.1559-1816.1997.tb01805.x

[B42] Lohmöller J. B. (1989). *Latent Variable Path Modeling with Partial Least Squares.* Berlin: Springer Science & Business Media 10.1007/978-3-642-52512-4

[B43] Lucidi F. Giannini A. M. Sgalla R. Mallia L. Devoto A. Reichmann S. (2010). Young novice driver subtypes: relationship to driving violations, errors and lapses. *Accid. Anal. Prev.* 42 1689–1696. 10.1016/j.aap.2010.04.008 20728618

[B44] Lucidi F. Mallia L. Lazuras L. Violani C. (2014). Personality and attitudes as predictors of risky driving among older drivers. *Accid. Anal. Prev.* 72 318–324. 10.1016/j.aap.2014.07.022 25108900

[B45] MacCallum R. C. Browne M. W. Sugawara H. M. (1996). Power analysis and determination of sample size for covariance structure modeling. *Psychol. Methods* 1 130 10.1037/1082-989X.1.2.130

[B46] Mallia L. Lazuras L. Violani C. Lucidi F. (2015). Crash risk and aberrant driving behaviors among bus drivers: the role of personality and attitudes towards traffic safety. *Accid. Anal. Prev.* 79 145–151. 10.1016/j.aap.2015.03.034 25823904

[B47] Martinussen L. M. Lajunen T. Møller M. Özkan T. (2013). Short and user-friendly: the development and validation of the Mini-DBQ. *Accid. Anal. Prev.* 50 1259–1265. 10.1016/j.aap.2012.09.030 23137991

[B48] Mattsson M. (2012). Investigating the factorial invariance of the 28-item DBQ across genders and age groups: an exploratory structural equation modeling study. *Accid. Anal. Prev.* 48 379–396. 10.1016/j.aap.2012.02.009 22664704

[B49] Mattsson M. (2014). On testing factorial invariance: a reply to JCF de Winter. *Accid. Anal. Prev.* 63 89–93. 10.1016/j.aap.2013.10.031 24275719

[B50] Mattsson M. Lajunen T. Gormley M. Summala H. (2015). Measurement invariance of the driver behavior questionnaire across samples of young drivers from Finland and Ireland. *Accid. Anal. Prev.* 78 185–200. 10.1016/j.aap.2015.02.017 25797304

[B51] Molnar L. J. Charlton J. L. Eby D. W. Langford J. Koppel S. Kolenic G. E. (2014). Factors affecting self-regulatory driving practices among older adults. *Traffic Inj. Prev.* 15 262–272. 10.1080/15389588.2013.808742 24372498

[B52] Molnar L. J. Eby D. W. (2008). The relationship between self-regulation and driving-related abilities in older drivers: an exploratory study. *Traffic Inj. Prev.* 9 314–319. 10.1080/15389580801895319 18696387

[B53] Moták L. Gabaude C. Bougeant J. C. Huet N. (2014). Comparison of driving avoidance and self-regulatory patterns in younger and older drivers. *Transp. Res. Part F* 26 18–27. 10.1016/j.trf.2014.06.007

[B54] Nasreddine Z. S. Phillips N. A. Bédirian V. Charbonneau S. Whitehead V. Collin I. (2005). The montreal cognitive assessment, MoCA: a brief screening tool for mild cognitive impairment. *J. Am Geriatr. Soc.* 53 695–699. 10.1111/j.1532-5415.2005.53221.x 15817019

[B55] Owsley C. Stalvey B. Wells J. Sloane M. E. (1999). Older drivers and cataract: driving habits and crash risk. *J. Gerontol. Series A* 54 M203–M211. 10.1093/gerona/54.4.M203 10219012

[B56] Özkan T. Lajunen T. Summala H. (2006). Driver behaviour questionnaire: a follow-up study. *Accid. Anal. Prev.* 38 386–395. 10.1016/j.aap.2005.10.012 16310749

[B57] Parker D. Reason J. T. Manstead A. S. Stradling S. G. (1995). Driving errors, driving violations and accident involvement. *Ergonomics* 38 1036–1048. 10.1080/00140139508925170 29105607

[B58] R Development Core Team. (2013). *R: A Language and Environment for Statistical Computing.* Vienna: R Foundation for Statistical Computing.

[B59] Reason J. Manstead A. Stradling S. Baxter J. Campbell K. (1990). Errors and violations on the roads: a real distinction? *Ergonomics* 33 1315–1332. 10.1080/00140139008925335 20073122

[B60] Rimmö P. A. (2002). Aberrant driving behaviour: homogeneity of a four-factor structure in samples differing in age and gender. *Ergonomics* 45 569–582. 10.1080/00140130210145873 12167200

[B61] Rimmö P. A. Hakamies-Blomqvist L. (2002). Older drivers’ aberrant driving behaviour, impaired activity, and health as reasons for self-imposed driving limitations. *Transp. Res. Part F* 5 47–62. 10.1016/S1369-8478(02)00005-0

[B62] Rosseel Y. (2012). Lavaan: an r package for structural equation modeling. *J. Stat. Softw.* 48 1–36. 10.18637/jss.v048.i02 25601849

[B63] Rowe R. Roman G. D. McKenna F. P. Barker E. Poulter D. (2015). Measuring errors and violations on the road: a bifactor modeling approach to the driver behavior questionnaire. *Accid. Anal. Prev.* 74 118–125. 10.1016/j.aap.2014.10.012 25463951

[B64] Schermelleh-Engel K. Moosbrugger H. Müller H. (2003). Evaluating the fit of structural equation models: tests of significance and descriptive goodness-of-fit measures. *Methods Psychol. Res. Online* 8 23–74.

[B65] Slavinskiene J. Žardeckaite-Matulaitiene K. Markšaityte R. Pranckevičiene A. Šeibokaite L. Endriulaitiene A. (2014). “Relations between traffic safety attitudes and self-reported risky driving in a sample of young traffic offenders,” in *Paper presented at the Transport Means - Proceedings of the International Conference*, 2014-January 289-292 (Washington, DC).

[B66] Smorti M. Guarnieri S. (2016). Exploring the factor structure and psychometric properties of the manchester driver behavior questionnaire (DBQ) in an Italian sample. *Test. Psychom. Methodol. App. Psychol.* 23 185–202. 10.4473/TPM23.2.4

[B67] Stephens A. N. Fitzharris M. (2016). Validation of the driver behaviour questionnaire in a representative sample of drivers in Australia. *Accid. Anal. Prev.* 86 186–198. 10.1016/j.aap.2015.10.030 26584016

[B68] Sullivan K. A. Smith S. S. Horswill M. S. Lurie-Beck J. K. (2011). Older adults’ safety perceptions of driving situations: towards a new driving self-regulation scale. *Accid. Anal. Prev.* 43 1003–1009. 10.1016/j.aap.2010.11.031 21376894

[B69] Ulleberg P. Rundmo T. (2003). Personality, attitudes and risk perception as predictors of risky driving behaviour among young drivers. *Safety Sci.* 41 427–443. 10.1016/S0925-7535(01)00077-7

[B70] Wong I. Y. Smith S. S. Sullivan K. A. (2015). The development, factor structure and psychometric properties of driving self-regulation scales for older adults: has self-regulation evolved in the last 15 years? *Accid. Anal. Prev.* 80 1–6. 10.1016/j.aap.2015.03.035 25841080

[B71] World Health Organization [WHO] (2015). *Global Status Report on Road Safety 2015.* Geneva: World Health Organization.

[B72] Yagil D. (1998). Gender and age-related differences in attitudes toward traffic laws and traffic violations. *Transp. Res. Part F* 1 123–135. 10.1016/S1369-8478(98)00010-2

[B73] Zeileis A. Kleiber C. Jackman S. (2008). *Regression Models for Count Data in R.* Available at: http://www.jstatsoft.org/v27/i08/

